# Whole-genome sequence analysis through online web interfaces: a review

**DOI:** 10.5808/gi.20038

**Published:** 2022-03-31

**Authors:** A. W. A. C. W. R. Gunasekara, L. G. T. G. Rajapaksha, T.L Tung

**Affiliations:** 1 Veterinary Medical Center and College of Veterinary Medicine, Jeonbuk National University, Jeonju 54596, Korea; 2 Department of Botany, Dagon University, 11422 Yangon, Myanmar

**Keywords:** average nucleotide identitys, online web servers, single nucleotide polymorphisms, virulence factors, whole-genome sequencing

## Abstract

The recent development of whole-genome sequencing technologies paved the way for understanding the genomes of microorganisms. Every whole-genome sequencing (WGS) project requires a considerable cost and a massive effort to address the questions at hand. The final step of WGS is data analysis. The analysis of whole-genome sequence is dependent on highly sophisticated bioinformatics tools that the research personal have to buy. However, many laboratories and research institutions do not have the bioinformatics capabilities to analyze the genomic data and therefore, are unable to take maximum advantage of whole-genome sequencing. In this aspect, this study provides a guide for research personals on a set of bioinformatics tools available online that can be used to analyze whole-genome sequence data of bacterial genomes. The web interfaces described here have many advantages and, in most cases exempting the need for costly analysis tools and intensive computing resources.

## Introduction

The development of DNA sequencing has revolutionized the idea of a genome and the knowledge of genes. These technologies have a dynamic history, which occurred within the last few decades. In brief, whole-genome shotgun techniques were first applied in 1979 for small size genomes ranging from 4000-7000 bp in experimental levels followed by a well-established DNA sequencing technique called “Sanger sequencing” which developed in the 1980s [1,2]. Rapid developments of the DNA sequencing techniques made it possible for automated sequencing in the 1990s, which allowed the first fully sequenced genome, *Haemophilus influenzae* in 1995 [3]. Later, around 2003 the sequencing of the entire human genome was completed [4,5]. Since then, numerous sequencing methods have been developed and they have evolved into a commercial platform called NGS or next-generation sequencing. Among many NGS technologies available, whole-genome sequencing (WGS) is involved with the determination of the entire DNA sequence from an organism’s genome at a single time [6]. It involves identifying the nucleotide arrangement of a complete genome of an organism, which is supported by automatic DNA sequencing methods and computational techniques that facilitates the assembly of millions of small DNA fragments [7]. Today, the advances and extensive use of NGS techniques have greatly affected the progress of the scientific research field.

Early WGS methods were expensive, difficult to perform, and time-consuming, especially in the developmental era of genomic data [8]. A decade ago, high-quality reference genome sequences were only available for a model or well-studied organisms. Today, the implementation of WGS facilitates a better understanding of the genomic functions in an organism and its expression mechanisms. Moreover, WGS provides much more comprehensive information on various genes by sequencing the noncoding DNA regions, which captures 95%–99% of the genome. The information gained through WGS has proven to be very useful in terms of understanding the origins of pathogenic microorganisms, their transmission routes, and in public health management [9,10]. Genome-wide approaches enhance the power and resolution for the above-mentioned applications and improve the reliability of conclusions.

There is no doubt that every WGS project needs a considerable cost and effort to address the questions at hand. However, the analysis of WGS data highly depends on sophisticated bioinformatics tools. Many laboratories and research institutions do not have the bioinformatics capabilities to analyze the large amount of genomic data generated through sequencing and therefore are unable to take maximum advantage of WGS [11]. The goal of this study is to provide a guide for research personals on bioinformatics tools available online that are needed to interpret WGS data and, how these online web interfaces can be applied to bacterial genome analysis settings easily, affordably, and, in most cases, without the need for intensive computing resources and infrastructure. Moreover, in this article, we discuss how to utilize genomic annotation servers, classical multilocus sequence typing (MLST), whole-genome MLST (wgMLST), single nucleotide polymorphisms (SNPs), average nucleotide identity (ANIs), prophages, cluster of orthologous groups (COG), virulence factors, and, genomic mapping tools for bacterial WGS data analysis. There is still much work that needs to be done for the development of online web interfaces to improve data quality and its applications in WGS. Consequently, it is necessary to develop more advanced and efficient data analysis pipelines for processing and analyzing whole genomes.

## General Workflow of WGS

**Fig. 1.**
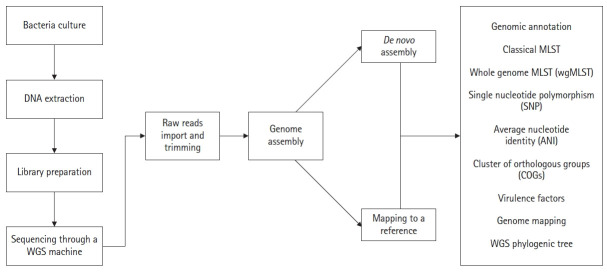
General overview of high throughput sequencing workflow of a bacterial genome. Following genome assembly, online web interfaces can be utilized for the purpose of analyzing WGS. MLST, multilocus sequence typing; WGS, whole-genome sequencing.

Several steps are involved in a bacterial WGS project. First, a biological sample (bacteria) is collected and cultured on appropriate media. The DNA is extracted by using commercial DNA extraction kits and/or by manual DNA extraction methods. The DNA quality is usually measured through the qubit meter. Following this, a DNA library is prepared. Once the DNA library is prepared, sequencing can be performed in any WGS machine (such as Illumina/ion torrent) as the researcher's requirements. Millions of short sequence reads are produced as the final result, typically a few hundred nucleotides long or less. After sequencing, raw reads will be trimmed to remove adopter and low-quality reads. By using these reads, the novel genome can be reconstructed with or without using a reference sequence. In reference-based reconstruction, the short reads are aligned to a closely related reference genome, which has a complete genomic representation. It is important to note that all the reads will not align with the reference genome (there can be some novel regions in the genome of interest that are absent in the reference genome). Sites with problematic nucleotide compositions also can be filtered out. As an alternative for reference mapping, *de novo* assembly can be performed. Here, all the short reads are aligned to each other (known as contigs) without the use of a reference sequence. The number of contigs produced depends on the total number of short-read DNA sequences in hand. Following reconstruction, the novel genomes can be analyzed through online web interfaces as described below (Fig. 1).

## The Genomic Annotation

**Fig. 2.**
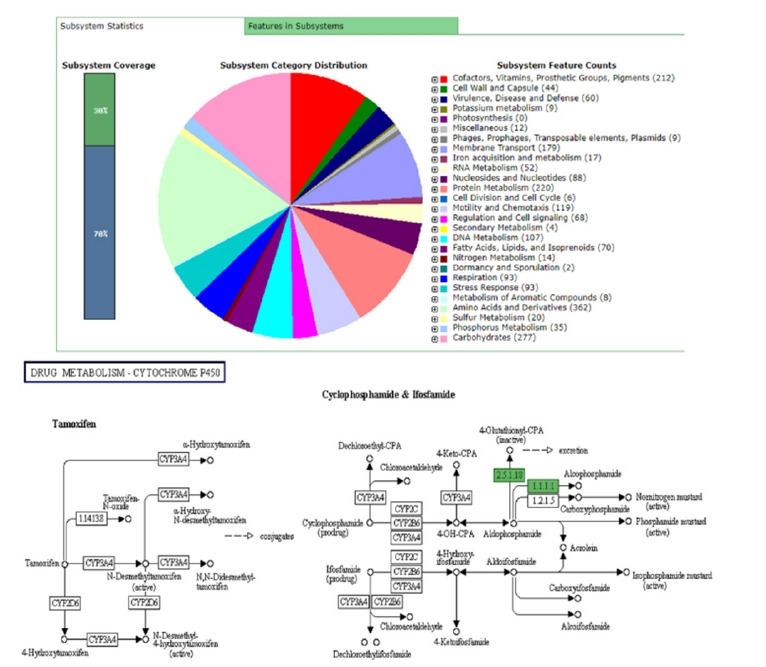
General subsystem features and KEGG pathway of drug metabolism of *Vibrio parahaemolyticus* 3HP_AHPND_ genome through RAST server (Different colors in the subsystem category distribution indicates different subsystem features whereas KEGG pathway indicates the functions for *V. parahaemolyticus* 3HP_AHPND_ genome). KEGG, Kyoto Encyclopedia of Genes and Genomes.

Once the assembly of a bacterial genome is completed, the next important step is genomic annotation. Simply it refers to the identification of functional/non-functional genomic segments and/or open reading frames and matching them to other reference genome sequences in an existing database [12]. A typical genomic annotation must include biological information such as gene models and gene functions and their protein products [13]. The annotation of a genome is depending on a set of rules guided by the annotation pipeline. Hence, the quality of the annotation always relies on the quality of the genome assembly [14]. Apart from the NCBI prokaryotic genome annotation server (PGAP), rapid subsystem annotation using subsystem technology or RAST annotation (http://rast.theseed.org/) is the most common pipeline available online for bacterial genome annotation [15]. Aside from subsystem statistics, the RAST annotation server is capable of providing metabolic construction along with functional, sequence, and KEGG database pathways (Kyoto Encyclopedia of Genes and Genomes database) through the annotation of a respective genome. Depending on the job load, annotation time for a genome can be varying. Final output data is available in various types of file formats which is very important for further analysis of genomes (Fig. 2).

## Classical MLST and SNP Calling

Correct, standardized identification is a basic need for any researcher working with bacteria, whether it’s a pathogen, commensals, or used for industrial purposes. For a long time, MLST has been considered as the “gold standard” for bacterial classification, and has been used widely for molecular studies [16]. Classical MLST or multilocus sequence typing is a technique that usually depends on seven housekeeping genes that reside in the bacterial genome [17]. The unique sequences of housekeeping genes in bacteria are assigned to a random integer number, in order to assign a unique genome profile (also known as allelic profile) which specifies its sequence type (ST). Since the ST is universal, the data collected through MLST has proven to be useful in characterizing bacterial isolates of different epidemiological origins [18]. To date, the PubMLST server is considered the most popular database on the internet related to MLST [19]. Finding a housekeeping gene sequence from bacterial WGS data can be time-consuming. Apart from PubMLST, various easy-to-handle online servers are available with the capability to identify classical MLST genes directly from a whole-genome sequence.

In a bacterial genome, analyzing SNP is considered as an important step in terms of understanding genomic relationships. The SNPs are the mirror showing how far your genome is divergent from other reference strains. In a typical bacterial genome, the presence of a small number of SNPs indicates that they are genetically similar and can be originated from a common ancestor [20]. Sometimes when isolates are distant in time or geographical origin, a large number of SNPs are present in between the respective genomes, indicating that they did not originate from the same source and/or they have been gone through evolution for a longer period [16]. Hence, the SNP base similarities and differences allow researchers to trace the transmission patterns of pathogenic organisms worldwide [21].

**Fig. 3.**
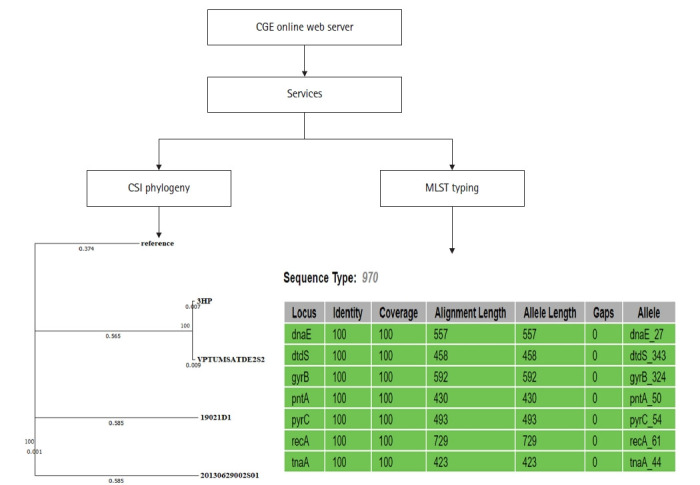
CGE server workflow of classical MLST typing and SNP calling on selected *Vibrio parahaemolyticus* genomes. (A) Clinical *V. parahaemolyticus* genome RIMD 221063 was used as a reference for SNP calling. (B) The *V. parahaemolyticus* 3HP_AHPND_ genome was used for the in silco MLST analysis. MLST, multilocus sequence typing; SNP, single nucleotide polymorphism.

The center for genomic epidemiology (https://cge.cbs.dtu.dk/services/) provides both classical MLST and SNP analysis of WGS. This server offers a comprehensible researcher friendly platform. The MLST scheme in the CGE server is associated with the PubMLST database [22]. Once the bacterial genomic data is uploaded to the server, each allelic number/their sequences representing housekeeping genes and ST can be obtained within a few minutes. The SNP analysis in the CGE server depends on a set of parameters selected by the user [23]. To analyze the SNP variations, it is necessary to upload the reference genome along with the genomes of interest. Most importantly this server is capable of producing an SNP base phylogenetic tree with evolutionary distances, and it is available in several file formats. As a result, users can modify the phylogenetic trees according to their requirements (Fig. 3).

## wgMLST and ANI Analysis

**Fig. 4.**
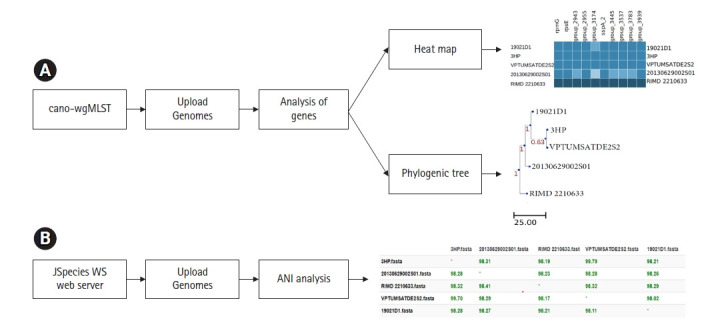
(A) The cano-wgMLST server workflow of wgMLST phylogeny and identification of highly discriminatory genes on 5 *Vibrio parahaemolyticus* genomes. (B) JSpeciesWS server workflow of ANI among 5 *Vibrio parahaemolyticus* genomes. ANI, average nucleotide identity; wgMLST, whole-genome multilocus sequence typing.

Many researchers suggested, previously mentioned classical MLST scheme doesn’t provide a higher resolution of bacterial genomes when compared to the large number of DNA sequences available in hand [10,24]. On this aspect extended versions of the classical MLST scheme have been developed. Besides, many researchers focus on identifying differences in genes present in bacterial genomes. Studying differences of genes is a key determinant to understanding virulence and pathogenicity among different bacterial strains [25-27]. The newly developed whole-genome MLST or wgMLST tools enable the recognition of genetic variations among bacterial pathogens with high accuracy [28,29]. The online web interface called cano-wgMLST (http://baccompare.imst.nsysu.edu.tw/index.php) can be used as a primary tool to identify the differences between genes and/or similarities among genomes (Fig. 4). This server provides a phylogenetic tree, heat map as well as the percentage of gene occurrence among respective genomes. The phylogenetic tree is constructed based on the core genome and highly discriminatory genes [29].

ANI or average nucleotide identity refers to the measurement of nucleotide level similarity between two or more genomes [30]. The ANIs exhibit genetic relatedness among bacterial strains. In the early days of genomic research, DNA-DNA hybridization is considered as the gold standard to compare nucleotide identities of bacterial genomes [31]. In parallel to the evaluation of genomic technologies, various software’s have been developed to assess the ANIs among bacterial genomes. The simplest tool that can use to calculate nucleotide level similarities is the JSpeciesWS online web server (http://jspecies.ribohost.com/jspeciesws/). The server measures the probability of multiple genomes belonging to the same species by pairwise comparisons of ANIs (Fig. 4). It is suggested that closely related bacterial species share a high rate of nucleotide similarities [32]. On researchers point, it is an important aspect since it provides capabilities to track epidemiological outbreaks [30]

## Virulence Factors, Prophages, and COGs

**Fig. 5.**
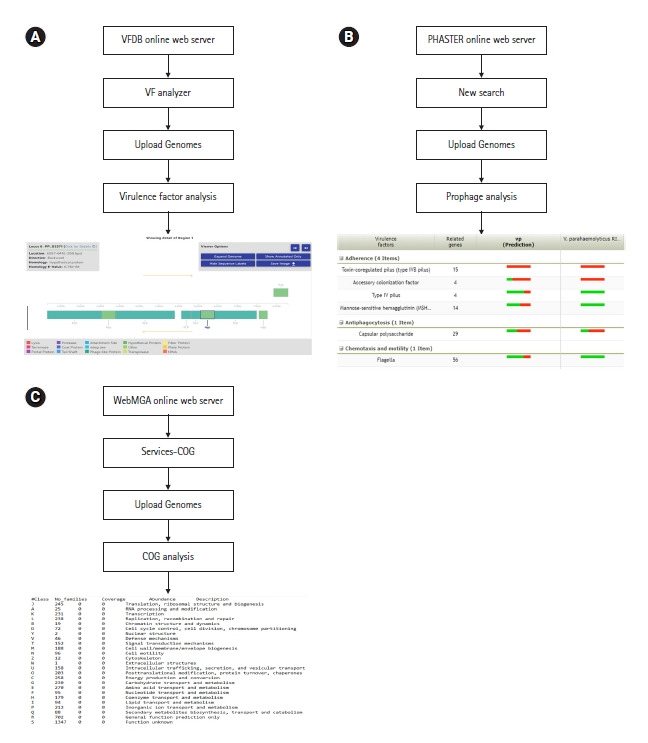
(A) Virulence factor analysis workflow of VFDB server. (B) Prophage analysis workflow of PHASTER server. (C) Cluster of orthologous group (COG) analysis workflow of WebMGA server. *Vibrio parahaemolyticus* genome 3HP_AHPND_ was used as a reference for all the applications.

Virulence factors are the properties of an organism that provide capabilities to establish itself on or within a particular host species and prompt the potential cause of the disease [26]. They are the driven forces of pathogenicity acquired by microorganisms, as a result of the long-term evaluation process. Common virulence factors of bacterial pathogens include adherence, anti-phagocytosis, chemotaxis and mortality, enzyme, iron uptake, quorum sensing, secretion systems, toxin, and immune evasion. Virulence factor database or VFDB is the most popular online server for bacterial genome-related virulence factor analysis (http://www.mgc.ac.cn/VFs/). This server allows the identification of virulence factors with structural features, mechanisms, and functions [33]. Furthermore, it is possible to analyze virulence factors in species level as well as the genus level through this server (Fig. 5A).

Prophages are the genetic materials that are inserted and integrated into bacterial chromosomes or plasmids without causing any disruption to the bacterial cell [34]. One key function of prophages is to increase the virulence potential of bacteria by horizontal gene transfer [35]. In terms of survival, prophages can give bacteria both resistance mechanisms and metabolic advantages [36]. The latest version of PHASTER (https://phaster.ca/) is an efficient, fast, and user-friendly online server in terms of prophage analysis [37]. The server provides graphical illustrations of prophages with their respective phage features. (Fig. 5B).

COG or cluster of orthologous groups is a set of proteins encoded by genomes of certain organisms related to direct evolution that are referred to be orthologous [38]. Studying COG in the recent past had a significant impact on the phylogenetic classification of proteins from microbial genomes [39]. The WebMGA (http://weizhong lab.ucsd.edu/webMGA/) is one web interface that predicts the COGs of bacterial genomes. The data will be available as a text file based on different COG classes. Following analysis, researchers can build graphical illustrations of COG as their requirements (Fig. 5C).

## Graphical Illustration of Genomes (Genome Mapping)

**Fig. 6.**
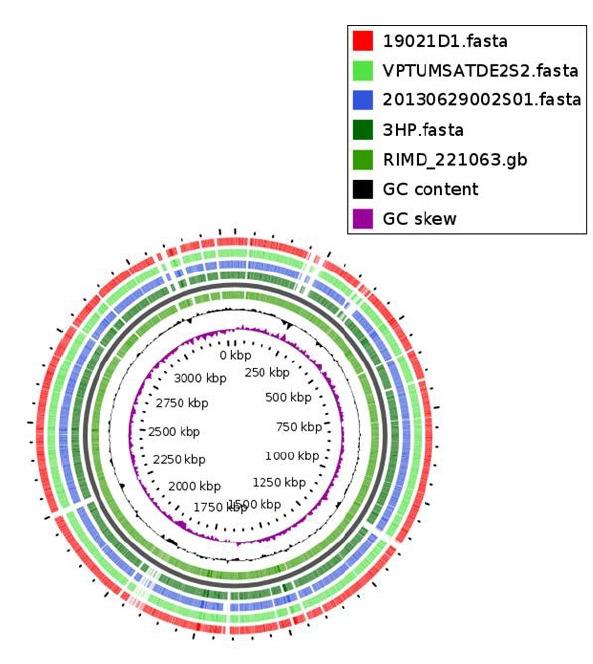
Graphical illustration of chromosome I for 5 *Vibrio parahaemolyticus* genomes by GView server. Different colors in the genome map indicate different genomes. Clinical *Vibrio parahaemolyticus* genome RIMD 221063 was used as the reference for the genomic mapping.

In general, genome mapping refers to the assignment of genes into their respective positions of the genomes [40]. To date, the majority of genomic mapping is conducted through highly sophisticated software. Difficulties of operating and high costs associated with the software lead many researchers to think twice when doing WGS projects. Several online servers are providing graphical illustrations of genomes. Representing genomic features is very important since they are the landmarks in the genome of an organism. It can effectively convey information that helps to understand the biological properties of microorganisms [41]. Also, unique information related to specific genes can be displayed in genomic maps. Furthermore, genomic maps can display sequence differences concerning a reference genome, gene expression, the positions of contigs for incomplete genomes, and the sequence coverage information. Among the limited number of online web servers developed so far, the CGview (http://stothard.afns.ualberta.ca/cgview_server/) and GView (https://server.gview.ca/) servers are widely used for graphical illustration of bacterial genomes. In these servers, parameters for a certain genomic map need to be set by the user. The CGview server provides a genomic map with distinct genomic features and through the GView server it is possible to analyze multiple genomes at once and generate a comparative genomic map (Fig. 6) [41].

Apart from the graphical illustrations of genomes, many researchers tend to use WGS based phylogenetic maps. The use of large-scale genomic data to generate a phylogenetic tree is impossible without analysis software and/or operating system. The WGS base phylogenetic trees lead researchers to understand evolutionary history and relationships among microorganisms [42]. There is a finite number of online servers available on this aspect. The CVTree3 (http://tlife.fudan.edu.cn/cvtree3/) is one such server that can be utilized for bacterial genomes in terms of phylogenetic tree mapping [43]. This web interface uses FAA or FFN files to produce phylogenetic trees. Annotation through the RAST server became very useful at this point. Because RAST server annotation provides FAA file as final output. The file generated through the RAST server is 100% compatible with the CVTree3. In this server, analysis of the genome highly depends on user-defined criteria. The phylogenetic trees generated through the CVTree3 server can be downloaded in various formats, which allows the researchers to modify them according to their requirements.

## Conclusion

Combined analysis of a respective genome along with ANI, SNPs, MLST, wgMLST, virulence, prophages, and COGs through these online web servers will motivate any researcher to move forward in bacterial WGS analysis without depending on other sophisticated genomic analysis tools. These web interfaces are deemed to be fast and accurate and can be used as a confirmation guide along with epidemiological analysis, research, and surveillance.
